# Non-invasive quantitative assessment of urethral compliance in rabbit tubularized incised plate model using ultrasound and uroflowmetry

**DOI:** 10.1038/s41598-025-11701-8

**Published:** 2025-07-20

**Authors:** Hongbo Liu, Wei Ru, Juan Zhou, Ciyuan Feng, Qibo Hu, Guangjie Chen, Weifeng Yang, Lizhe Hu, Xiang Yan

**Affiliations:** 1 Department of Urology, Children’s Hospital, Zhejiang University School of Medicine, National Clinical Research Centre for Child Health, Hangzhou, China; 2 Department of Ultrasound, Children’s Hospital, Zhejiang University School of Medicine, National Clinical Research Center for Child Health, Hangzhou, Zhejiang China

**Keywords:** Urethra, Compliance, Hypospadias, Ultrasonography, Uroflowmetry, Hypospadias, Urethra

## Abstract

Low urinary flow rates are frequently observed following tubularized incised plate urethroplasty. The underlying cause is not yet fully understood and may be associated with low urethral compliance. The purpose of this study is to non-invasively evaluate the urethral compliance in a rabbit model. Ten male New Zealand rabbits were randomly divided into a control group and a urethroplasty group for tubularized incised plate urethroplasty. Seven weeks post-operatively, ex vivo urethral compliance was evaluated using both the Jesus invasive measurement and the non-invasive method. The Jesus measurement involved measuring urethral volume and pressure by air insufflation, and the non-invasive method utilized ultrasound and uroflowmetry to assess the urethral anterior-posterior diameter and flow data, respectively. Curve regression analysis was applied to calculate urethral compliance. Curve regression analysis revealed that the median urethral compliance measured by non-invasive method in the control group was 0.247 (0.241, 0.257) mm•s/ml, and it was 0.269 (0.263, 0.270) mm•s/ml in the urethroplasty group, with no significant difference between the two groups. The Jesus method indicated median urethral compliance was 0.141 (0.137, 0.149) ml/cmH_2_O for the control group and 0.182 (0.173, 0.192) ml/cmH_2_O for the urethroplasty group, showing no significant statistical difference. In the rabbit model, urinary flow rate and anterior-posterior diameter serve as non-invasive indicators that can effectively reflect urethral compliance, and TIP surgery has no significant impact on urethral compliance.

**Supplementary Information:**

The online version contains supplementary material available at 10.1038/s41598-025-11701-8.

## Introduction

Hypospadias is a common congenital disorder of the male genitalia. Its overall prevalence is 1 in 300 to 1 in 200 live births and is on the rise^1,2^. The clinical manifestations include abnormal location of the urethral meatus, with some cases accompanied by penile curvature, which severely affects the patient’s urinary function and reproductive health. The main treatment for hypospadias is surgical reconstruction of the urethra, aiming to position the urethral meatus as close to the normal anatomical location as possible, thereby restoring urinary function and improving appearance. Common surgical methods include the Tubularized Incised Plate (TIP) urethroplasty, Mathieu urethroplasty using perimeatal flaps, Onlay urethroplasty using a transverse island pedicle flap, and Duckett urethroplasty using a transverse preputial island flap. The TIP technique involves degloving the penis and longitudinally incising the urethral plate, followed by the insertion of a urethral catheter and tubularization of the urethra to form a new urethra^3^. Due to its simplicity, low complication rate and good cosmetic result after the surgery^4^, the TIP technique is widely used in the repair of hypospadias.

However, uroflowmetry showed that asymptomatic reduced flow is common after TIP urethroplasty, despite the absence of anatomical urethral obstruction in most cases^5,6^. A low urinary flow rate can result in prolonged urination time and increased urine retention in the bladder, elevating the risk of urinary tract infections and potentially contributing to the formation of bladder stones. Some researchers believe that the decrease in urethral compliance, which makes it difficult for the urethra to expand during urination and thus hinders the smooth outflow of urine, leading to a reduced urinary flow rate. The decrease in urethral compliance may be attributed to the anatomical characteristics of hypospadias itself or the influence of the TIP surgical technique^7,8^.

The current assessment of urethral compliance is still an area of ongoing experimentation. Most studies rely on invasive methods and it is difficult to quantify the value of the urethral compliance^9–13^. However, for children with hypospadias, invasive procedures increase the risk of urethral injury, infection, pain and also increase parents’ concerns. It is of great value to seek a non-invasive and suitable method for evaluating urethral compliance in children.

The rabbit urethra is highly similar to the human urethra in terms of anatomical structure, epithelial tissue, muscle tissue, distribution of neurotransmitter receptors, and urinary control, and thus is widely used in research on urethral biomechanics^14^. In this study, we developed a novel non-invasive method for urethral compliance assessment using uroflowmetry and ultrasound of urethral diameter (Uroflowmetry and Diameter, UFD). The aim of this study was to evaluate the effectiveness and accuracy of the non-invasive UFD method and to investigate the impact of the TIP technique on urethral compliance in rabbit model.

## Materials and methods

### Study design

This study is designed as an equivalence randomized controlled trial. The healthy male New Zealand rabbits at 6 to 7 months of age weighing around 3 kg obtained from Yuhang KeLian Rabbitry (Hangzhou, China) were included in the study and randomly divided into two groups: the control group and the TIP group. Random numbers were generated using the standard = RAND() function in Microsoft Excel. Rabbits in both groups underwent urethral compliance assessment using the non-invasive UFD and invasive Jesus methods. The evaluators were cognizant of the grouping arrangements during both the allocation process and the outcome assessment phase. The surgeon, ultrasound physicians, uroflowmetry technicians, and data analysts were all unaware of the group assignments until the completion of the data analysis.

The study was completed in Zhejiang Chinese Medical University Laboratory Animal Research Center after receiving permission from Animal Ethical and Welfare Committee of Zhejiang Chinese Medical University (Ethics approval number: IACUC-20230529-08). Each rabbit serves as an individual experimental unit monitored in separate cages in a room with temperature 21 ± 1℃ and humidity of 40-70%. The surgery and measurement procedures for the rabbits were both completed within a single day. All procedures were conducted in accordance with the US National Institutes of Health (NIH publication no.85 − 23, revised 1996) and the ARRIVE guidelines.

### Sample size

Based on the preliminary results, the difference between UFD and Jesus groups is 0.08, with a standard deviation of 0.07. A two-sided α of 0.05 and a test power of 0.9 are used. The equivalence margin is 0.2, and the sample size ratio is 1:1. Using Chow et al.’s method^15^, and R 4.4.0 for calculations, 8 samples are needed per group. Considering a 10% surgical failure rate, and additionally, one extra experimental rabbit per group for histological staining analysis, combined with the fact that this study is a self-controlled experiment, the total sample size is 10 experimental rabbits (Table 1).

**Table 1:** Distribution of TIP and control groups across UFD, jesus, and pathological categories.

	UFD (*n* = 8)	Jesus (*n* = 8)	Pathological (*n* = 2)
TIP (*n* = 5)	4	4	1
Control (*n* = 5)	4	4	1
Total (*n* = 10)	8	8	2

UFD: uroflowmetry and diameter in ultrasound; TIP: tubularized incised plate.

### Surgical Procedures

Prior to the commencement of the experiment, all animals were provided with a one-week acclimation period to ensure their adaptation to the environmental conditions of the experimental facility. The TIP urethroplasty was performed on the TIP group to establish the TIP model by a surgeon with 20 years of surgical experience.

**Fig. 1:**
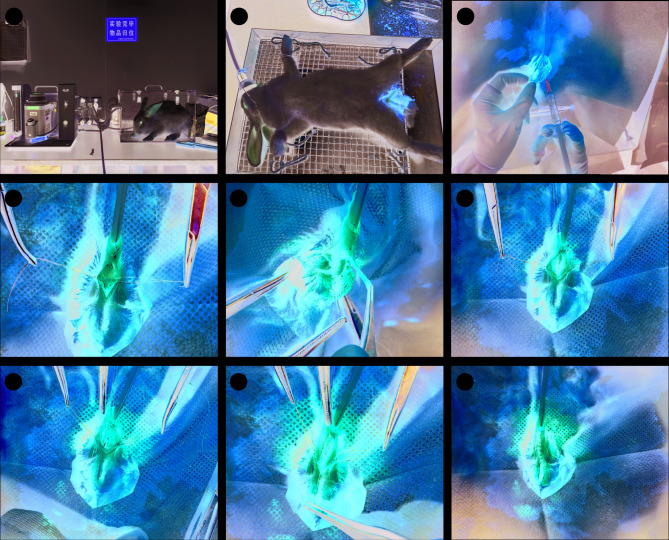
Step-by-step description of TIP procedures. (**A**) Induction of anesthesia; (**B**) Post-induction immobilization and maintenance of anesthesia; (**C**) Dorsal penile block; (**D**) Excision of the ventral urethral surface; (**E**) Exposure of the ventral urethra; (**F**) Status post-excision of the ventral urethra; (**G**) Longitudinal dorsal incision of the urethral plate; (**H**) Appearance after ventral urethral closure; (**I**) Appearance following full-thickness urethral suture.

TIP group: The animals placed supine and anesthesia induced with 5% isoflurane inhalation for 5 min, followed by anesthesia maintained with 2% isoflurane inhalation. Meanwhile, local dorsal penile nerve block anesthesia was performed by subcutaneous injection of 2 ml of lidocaine and ropivacaine mixed in a 2:1 ratio before surgery. After disinfection, 5 − 0 polypropylene was suture in the glans to provide traction, and a 10 Fr silicone urethral catheter was inserted. After longitudinal ventral incision of prepuce, the ventral half of the urethral was excised from 0.5 to 3 cm distance to meatus, creating a defective urethra as functionally hypospadias. A longitudinal midline dorsal incision was made on the opening urethral plate. Then ventral opening urethra was tubularized and urethroplasty was carried out with running suture using 6 − 0 polydioxanone, followed by subcutis and skin closure. A 10 Fr silicone urethral catheter was left indwelling without penile dressing (Fig. 1). The animals received routine wound disinfection care after recovering from anesthesia. Intramuscular penicillin sodium 100,000 iU/kg was required postoperatively, once daily for three days. Postoperatively, the urethral healing of the experimental animals is monitored daily.

Control group: The animals received routine care and daily monitoring of their overall health status. No any urethroplasty procedures was performed.

The animals both in TIP and control groups were sacrificed 7 weeks postoperatively. All animals were euthanized using 20 ml of air following anesthesia induction by 5% isoflurane. The corpus cavernosum and urethra was harvested from 5 cm distance to meatus after degloving (Supplements S1).

### UFD non-invasive urethral compliance assessment

#### Theoretical basis

The compliance of urethra (CUA) is defined as the slope of the pressure-volume relationship and can also be approximated by the change in diameter under pressure changes^16^. The formula is CUA = ΔV/ΔP ≈ ΔD/ΔP, where V represents urethral volume (ml), P represents urethral pressure (cmH₂O), and D represents the anteroposterior diameter of the urethra (mm). The anteroposterior diameter of the urethra is measured using ultrasound (Esaote’s MyLab™ Eight eXP, China), with the measurement location ranging from 0.5 to 3 cm proximal to the urethral meatus (the range for TIP urethroplasty model). According to Bernoulli’s equation and preliminary experimental data, the urethral pressure and urinary flow rate are linearly related (Supplements S2 and S3). Therefore, the formula is obtained as CUA_UFD_ = ΔD/ΔQ, where Q represents the urinary flow rate (ml/s).

#### UFD device

**Fig. 2:**
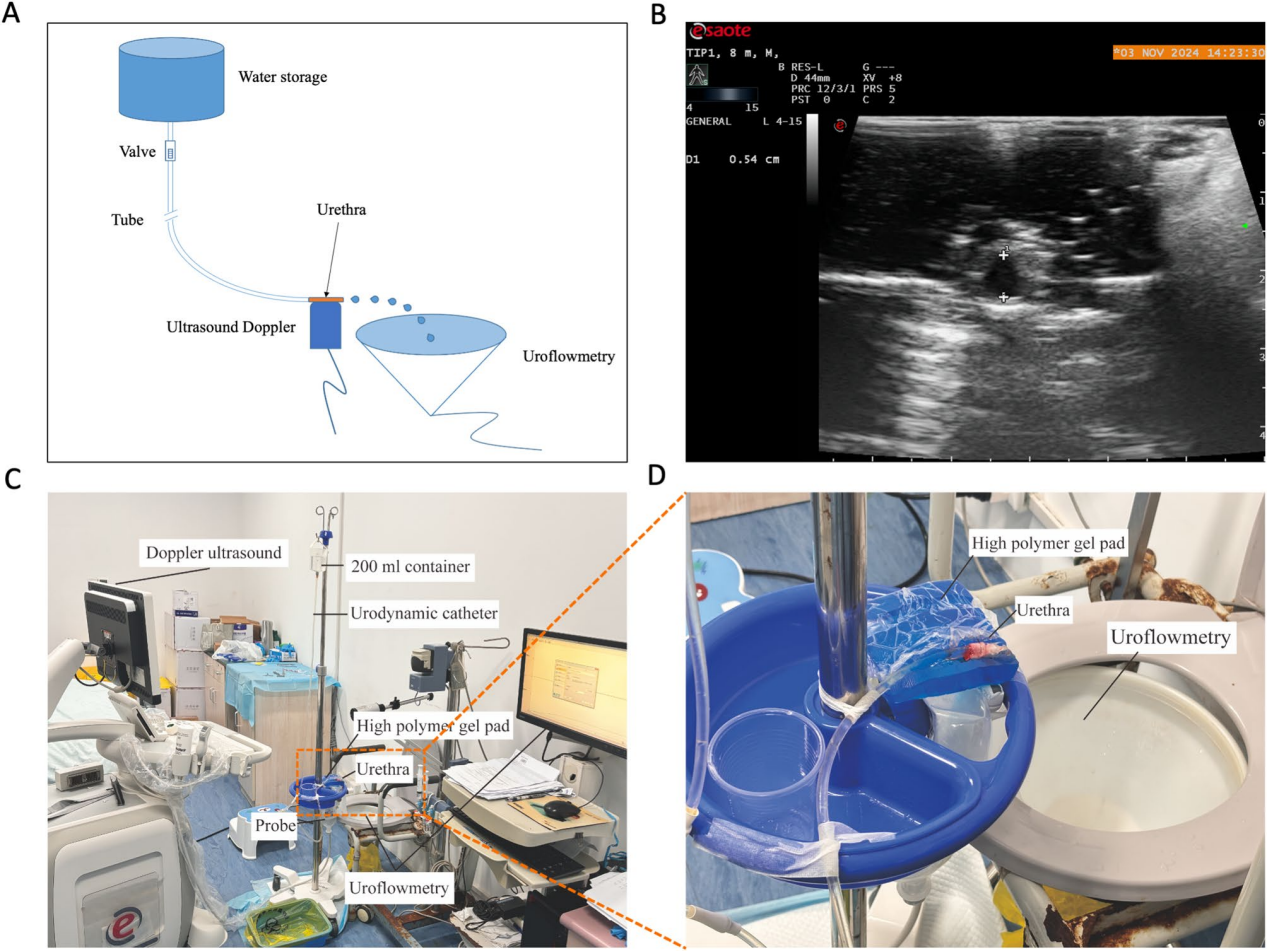
UFD non-invasive urethral compliance assessment device. (**A**) Schematic diagram of the device; (**B**) Ultrasound measurement of the anteroposterior diameter of the urethra; (**C**) Photograph of the actual device; (**D**) Photograph of the voiding component of the device.

The UFD device includes a container holding 200 ml of urine, which is connected to the urethra via a tube, and the bladder pressure changes are simulated by adjusting the height of the container. The bladder pressure during the rabbit’s storage phase is 10–30 cmH₂O, and micturition usually begins when the bladder pressure exceeds 40 cmH₂O. Due to considerable heterogeneity among the parameters studied and their observed values, reported maximum voiding pressures range from 70 to 160 cm H₂O^17^. Since rabbits have a urinary control mechanism similar to that of humans, the maximum height of the urine container is set at 100 cm above the urethra to avoid overdistension of the urethra. Therefore, the height range of the urine container in the UFD device is 40–100 cm above the urethra, which simulates the urethral expansion during the process of changing from low to high bladder pressure (Fig. 2). The UFD assessment should be performed within 30 min after the urethra is excised to prevent dehydration of the urethral tissue, and each measurement should not exceed 5 min. After all samples have undergone the UFD assessment, the invasive compliance assessment is carried out in sequence according to the random sequence generated by the standard = RAND()function in Microsoft Excel.

#### Jesus invasive urethral compliance device

**Fig. 3:**
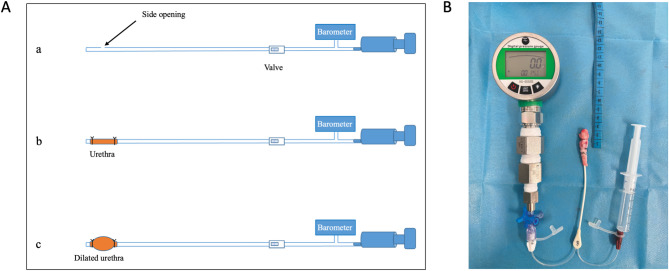
Invasive urethral compliance assessment device. (**A**) Schematic diagram of the device; (**B**) Photograph of the actual device.

The Jesus invasive urethral compliance assessment device consists of a 6 Fr urethral pressure measurement catheter, a precision digital pressure gauge (Nanjing Dedu Instrument Co., Ltd., China), and a 5 ml syringe. The pressure gauge and the urethral catheter are connected by a three-way tube. During measurement, the 6 Fr urethral catheter is inserted into the urethral tissue, and the ends of the urethra are ligated to seal it, leaving only a 1 cm measurement area (Fig. 3). When air is injected into the urethral cavity through the syringe, the intracavity pressure increases, and the urethral compliance can be calculated based on the changes of urethral volume and the pressure value (PSI), the formula is CUA_Jesus_ = ΔV/ΔP. The urethral volume can be calculated based on the ideal gas equation (**Supplements S4**). The Jesus measurement is performed immediately after the UFD measurement, with each urethral specimen measurement taking no more than 3 min.

### Pathology

One urethral specimen was randomly selected from both the control group and the TIP group for histological staining. These specimens were preserved in phosphate-buffered formalin and sectioned (with a thickness of 4 mm). Two sections from each animal were stained with Hematoxylin-Hosin staining (HE, for routine histological evaluation) and Picrosirius Red staining, respectively. Picrosirius Red staining was used to assess collagen deposition. The stained sections were photographed using Qupath software (open-source, UK). At a magnification of 10×, eight regions of interest (ROI) were selected in the urethral mucosa and submucosa for collagen fiber counting: ROIs 1 and  5 were the ventral midline, ROIs 2,3, 6 and 7 were the sides, and ROIs 4 and 8 were the dorsal midline. ImageJ software (National Institutes of Health, USA) was used for ROI quantitative analysis to evaluate the distribution of collagen fibers, with the main parameter being the percentage of collagen fiber area (%Area).

#### Statistics

The statistical analysis was performed using SPSS 26.0 (SPSS Inc, Chicago, IL) and R4.4.0. The normality of the data was tested using the Shapiro-Wilk test. Non-normally distributed continuous variables were expressed as median with interquartile range (IQR), and comparisons between groups were made using the Mann-Whitney test. Continuous variables that follow a normal distribution are described as means with standard deviations (Mean ± SD) and compared using the Student’s t-test. Curve regression is used to reflect the relationship between ΔD and ΔQ, as well as ΔV and ΔP. The association between the two methods was evaluated by Spearman correlation analysis, and their agreement was visualized with Bland–Altman plots. A *P*-value of less than 0.05 was considered significant.

## Results

### Descriptive information

The baseline body weights in the control group and the TIP group were 3.11 (2.93, 3.29) kg and 3.12 (3.00, 3.24) kg, respectively, with no statistically significant difference between the two groups. No rabbit developed postoperative urethroplasty related complications and death.

### UFD and Jesus urethral compliance assessment (*n* = 8)

**Fig. 4:**
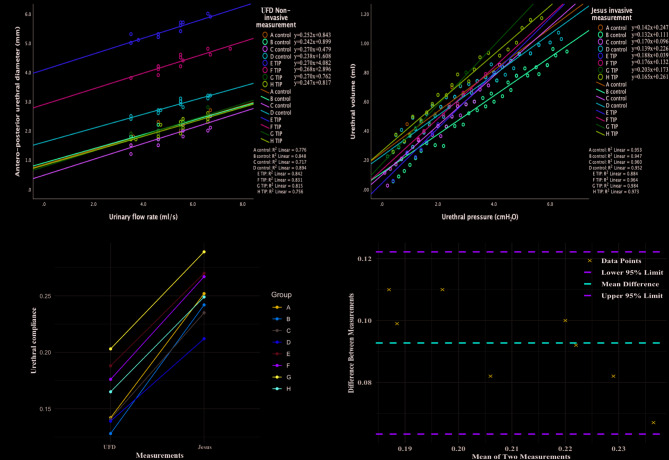
Fitting curves and correlation and consistency analysis of UFD and Jesus assessments. (**A**) Fitting curves of the control and TIP groups in the UFD assessment; (**B**) Fitting curves of the control and TIP groups in the Jesus assessment; (**C**) Correlation analysis between the two measurements. (**D**) Bland-Altman plot, consistency analysis between the two measurements.

Curve regression results show that the UFD median urethral compliance in the control group and the TIP group were 0.247 (0.241, 0.257) mm•s/ml, and 0.269 (0.263, 0.270) mm•s/ml, respectively, with no statistically significant difference between the two groups (*P* = 0.237). The Jesus median urethral compliance for the control group and the TIP group were 0.141 (0.137, 0.149) ml/cmH_2_O, and 0.182 (0.173, 0.192) ml/cmH_2_O, respectively. There was no statistically significant difference between the two groups (*P* = 0.057) (Fig. 4).

### Correlation and consistency analysis (*n* = 8)

The correlation analysis revealed a strong association between urethral compliance values measured by UFD method and Jesus method (Spearman’s ρ = 0.878, *P *= 0.004). Paired line plots further demonstrated good within-subject correlation. Bland–Altman analysis confirmed good agreement between the two measurements. (Fig. 4).

### Pathology results (*n* = 2)

**Fig. 5:**
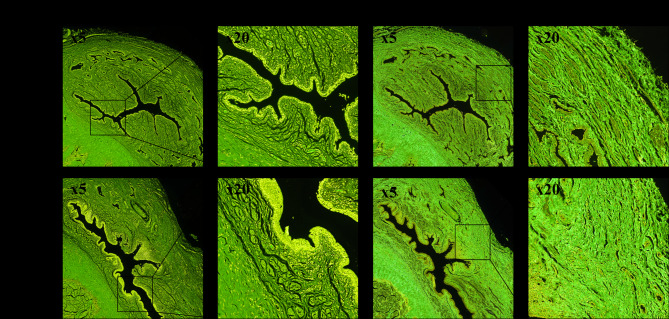
Urethral tissue histological staining. Control group: Hematoxylin and eosin (HE) staining shows that the urethral epithelium is composed of three layers of cells, with a clear urethral lumen and intact epithelium, submucosa, muscular layer, and vascular sinus. Picrosirius Red staining shows well-arranged muscle fibers. TIP group: HE staining shows intact urethral epithelium composed of 4 – 7 layers of epithelial cells. At the dorsal incision site, the submucosa is absent, and there is a lack of longitudinal muscle fibers and vascular sinus. Picrosirius Red staining shows inflammatory cell infiltration in the sutured area, with disordered muscle fiber arrangement and mild congestion.

Compared to the control group, seven weeks after TIP surgery, complete epithelial healing was observed, with evidence of epithelial cell hyperplasia and no luminal narrowing. Lymphocytic infiltration was present in the surgical area, accompanied by the absence of the submucosal layer, and a lack of longitudinal muscle fibers and vascular sinuses. The muscle fibers were disorganized and slightly dense, with no signs of scar formation (Fig. 5).

**Fig. 6:**
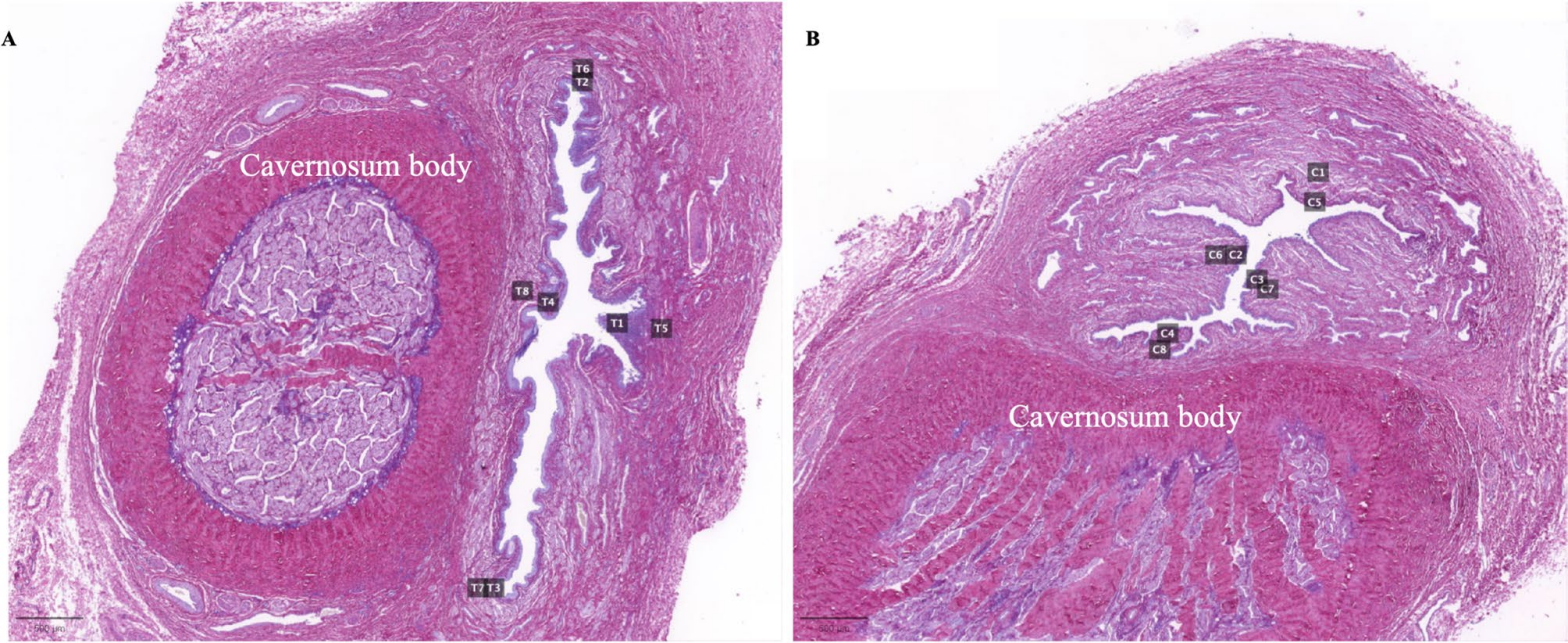
Regions of Interest in urethral tissue sections of the TIP group and control group. (**A**) TIP group; (**B**) Control group.

The quantitative analysis results of Picrosirius Red staining (Fig. 6) showed that the mean percentage of collagen fibers in the TIP group was 35.60% ± 8.70%, while in the control group, it was 32.20% ± 12.05%. The difference between the two groups was not statistically significant (*P* = 0.53). Within the TIP group, the percentage of collagen fibers in the mucosa was 35.10% ± 3.20%, and in the submucosa, it was 36.11% ± 12.87%. The difference between these two layers was not statistically significant (*P* = 0.89). In the control group, the percentage of collagen fibers in the mucosa was 34.86% ± 15.88%, and in the submucosa, it was 29.54% ± 8.23%. The difference between these two layers was also not statistically significant (*P* = 0.58) (Table 2).

**Table 2:** Analysis of intergroup and intragroup differences in collagen fiber area proportion (Collagen % Area).

Group	Layer	Mean ± SD	*P*-value
TIP group	-	35.60 ± 8.70	0.53
Control group	-	32.20 ± 12.05	
TIP group	Mucosa	35.10 ± 3.20	0.89
	Submucosa	36.11 ± 12.87	
Control group	Mucosa	34.86 ± 15.88	0.58
	Submucosa	29.54 ± 8.23	

## Discussion

Decreased urethral compliance has previously been considered one of the causes of low urinary flow rate after hypospadias repair. In this study, a novel non-invasive UFD method for quantitative urethral compliance assessment was developed based on urinary flow rate and Doppler ultrasound. Rabbit models were conducted to validate its high consistency with the invasive Jesus assessment method. Moreover, the results of both the Jesus measurement and the UFD measurement showed that TIP surgery had no significant impact on urethral compliance.

Several studies have reported methods for measuring urethral compliance. Walter et al.^9^ proposed a method in 1994 to assess the male urethral area equivalent factor (AEFm) through detrusor pressure and urinary flow rate, and then assess urethral compliance through the ratio of AEFm to detrusor pressure. However, this method requires invasive urodynamic examination to obtain detrusor pressure and is not well-suited for pediatric patients due to poor cooperation during urodynamic examinations. Lalla et al.^11^ suggested using scanning acoustic microscopy to measure the biomechanical and histological properties of the urethra, but this method is destructive to the urethra and is only suitable for use on excised urethral segments. Faurschou et al.^13^ used the EndoFLIP^®^ catheter system to measure urethral biomechanical properties, which includes a non-compliant balloon filled with conductive solution to measure the diameter of different urethral segments. By incrementally increasing the balloon pressure from 40 cmH_2_O and 60 cmH_2_O and recording the expansion and deformation of the urethra under pressure, urethral compliance is assessed based on the time/diameter curve. However, the EndoFLIP^®^ catheter system is primarily used for esophageal or cardia diseases, and its catheter size is too large for children’s urethra. The Jesus method assesses urethral compliance by inflating the urethra and measuring the intraluminal pressure^12^. It is highly reproducible and accurate but is destructive to the urethra. In summary, previous studies were invasive. Therefore, this study proposes a non-invasive method for assessing urethral compliance for potential clinical use. In clinical practice, we can measure the changes in the anteroposterior diameter of the urethra and the urinary flow rate before and after the patient voids normally and then strains, to calculate urethral compliance. Given the feasibility and high accuracy of the Jesus assessment method, this study, based on the invasive urethral compliance measurement by Jesus, quantifies the urethral compliance value through curve fitting of urethral volume and intraluminal pressure. This serves as a validation of the accuracy of the UFD method.

The UFD method innovatively combines two commonly used non-invasive clinical methods to assess urethral compliance. Urinary flow rate, which evaluates urinary function based on the volume of urine passing through the urethra, is widely used in clinical practice. Preliminary experiments have shown a linear correlation between urinary flow rate and urethral pressure, which can be used to reflect urethral pressure. Ultrasound, due to its convenience and lack of radiation, is often used as a diagnostic tool in pediatric patients and has high accuracy in measuring the anteroposterior diameter of the urethra. Under dynamic pressure increase, urethral compliance is assessed by combining the changes in the anteroposterior diameter of the urethra. In the fitting curve of urethral anteroposterior diameter versus urinary flow rate, the slope represents urethral compliance. Since the model used in this study is non-urethral obstructive, it is only applicable to cases without urethral obstruction.

After TIP surgery, urethral epithelialization is achieved through the re-coverage of the exposed epithelial layer, which facilitates wound healing. The status of urethral epithelialization is an important indicator for assessing the effectiveness of healing^18^. Previous studies have shown that acute tissue reactions subside after 6 weeks^19^. To ensure that the acute reaction has completely resolved, we chose to harvest the urethra at 7 weeks postoperatively and found complete epithelialization of the urethra, which is consistent with previous studies^20,21^. However, chronic inflammatory reactions still exist at 7 weeks postoperatively, which is in line with the research findings of Genç and Hafez^22,23^. Eassa et al.^24^ assessed pathological changes during the healing process after TIP surgery in a rabbit model, showing complete urethral healing without fibrosis or scar formation two weeks postoperatively. In canine and porcine models, no excessive collagen deposition or scar formation was observed 21 days after TIP surgery^25,26^. Previous studies have shown that urethroplasty can lead to tissue fibrosis and the formation of secondary urethral stricture^27^. However, the results of this study indicate that no significant fibrosis or scarring was observed after TIP surgery. Additionally, the quantitative assessment of urethral compliance revealed that TIP surgery is not a cause of decreased urethral compliance. However, the presence of chronic inflammatory reactions may have an impact on the results.

This study has several limitations. First, the rabbit model did not replicate primary hypospadias but rather a surgically induced form. It is well established that the urethral plate in patients with hypospadias exhibits distinct histological characteristics compared to individuals with a normal urethra. These differences could explain the lack of observed differences between groups in terms of urethral compliance and fibrosis after the surgery. This study can only demonstrate that TIP surgery had no significant impact on urethral compliance in the rabbit hypospadias model, but it cannot indicate the impact in patients with hypospadias. Further investigation in these patients is needed. Second, the study used an ex vivo urethral model that was not in a physiological state. It could not fully simulate the performance of the urethra in vivo under normal physiological conditions with blood perfusion and neural control. The biomechanical behavior of the urethra may differ due to the absence of these factors. Future studies could assess urethral compliance in vivo using non-invasive compliance assessment methods. Finally, the study measured urethral compliance at 7 weeks after TIP surgery in rabbits and only concluded that no significant decrease in compliance was found in the short term after surgery. The pathological results at 7 weeks postoperatively still showed chronic inflammatory reactions, which may be related to the long-term exposure of the urethra to the urine environment and the tissue repair process. Future research could extend the follow-up period to explore the changes in urethral compliance after complete recovery.

## Conclusion

This study developed and validated a non-invasive method for calculating urethral compliance based on urinary flow rate and the anteroposterior diameter of the urethra. It was found that in the rabbit hypospadias model, TIP surgery had no significant impact on urethral compliance.

## Electronic supplementary material

Below is the link to the electronic supplementary material.

Supplementary Material 1

## Data Availability

Data are available from the corresponding author on reasonable request.
