# Supplementary material for: Non-invasive quantitative assessment of urethral compliance in rabbit tubularized incised plate model using ultrasound and uroflowmetry
Source: Sci Rep. 2025 Jul 20;15:26331. doi: 10.1038/s41598-025-11701-8 (PMC12277407; doi:10.1038/s41598-025-11701-8)
Supplement: Supplementary file 1 — Supplementary Material 1 [file 41598_2025_11701_MOESM1_ESM.pdf]

Supplement materials S1

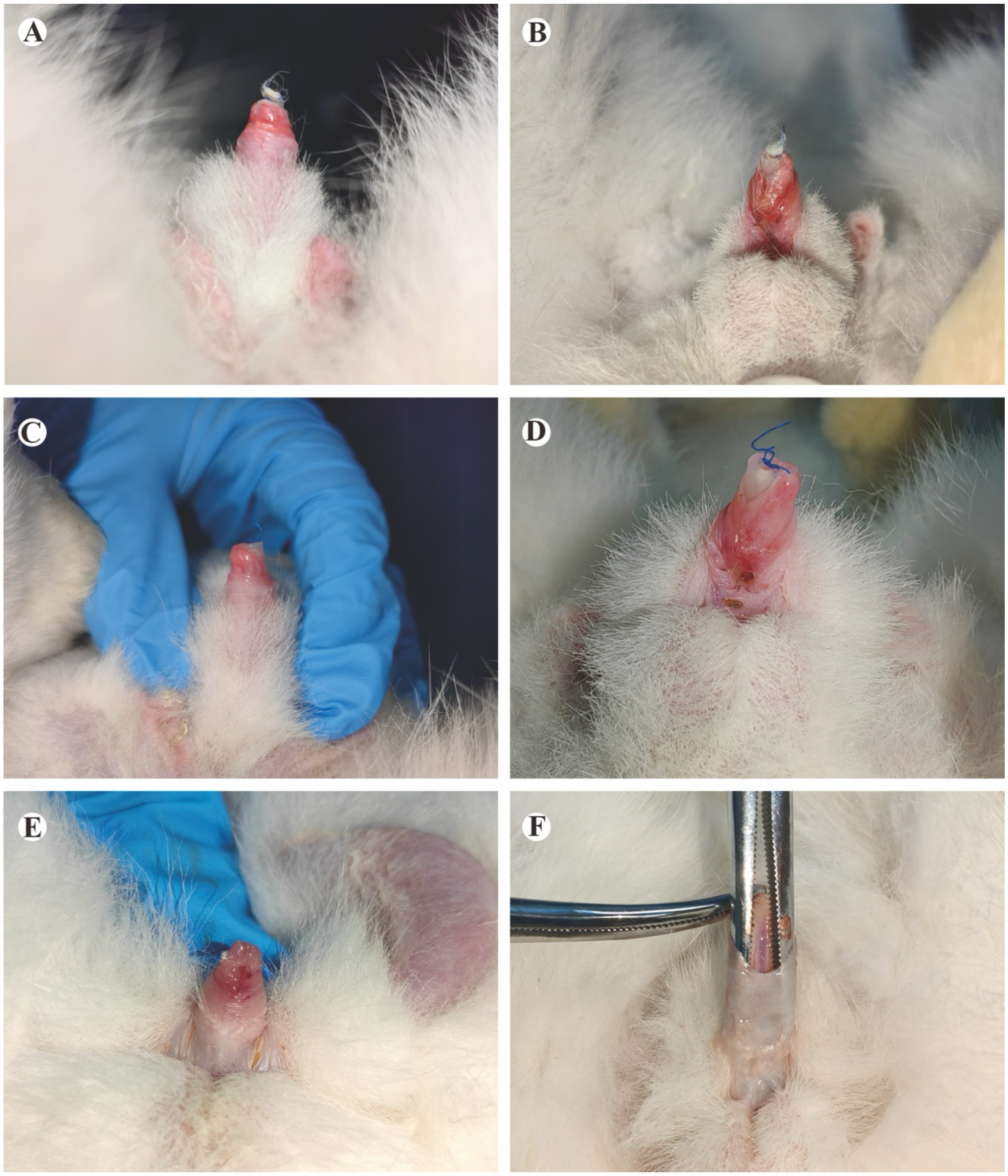

Fig.S1. Postoperative urethral recovery following TIP surgery. A-B. Three days postoperatively; C-D. Seven days postoperatively; E-F. Seven weeks postoperatively.

Supplement materials S2. Derivation of urethral pressure calculation in non-invasive measurement (no figure included)

According to Bernoulli's equation, $P + \frac{1}{2}\rho v^2 + \rho gh = c$, where P is the pressure of the fluid, ρ is the fluid density, v is the velocity, measured by ultrasound, g is the acceleration due to gravity, h is the height of the liquid, and c is a constant.

Taking any measurement point 1 in the urethra and the urethral outlet point 2, we get: $P_1 + \frac{1}{2}\rho v_1^2 + \rho gh_1 = P_2 + \frac{1}{2}\rho v_2^2 + \rho gh_2$.

Since the heights of the two points are essentially the same, we have $P_1 + \frac{1}{2}\rho v_1^2 = P_2 + \frac{1}{2}\rho v_2^2$.

Since the pressure at the urethral outlet point is approximately zero, we get $P_1 + \frac{1}{2}\rho v_1^2 = \frac{1}{2}\rho v_2^2$, which leads to $P_1 = \frac{1}{2}\rho(v_2^2 - v_1^2)$.

Supplement materials S3

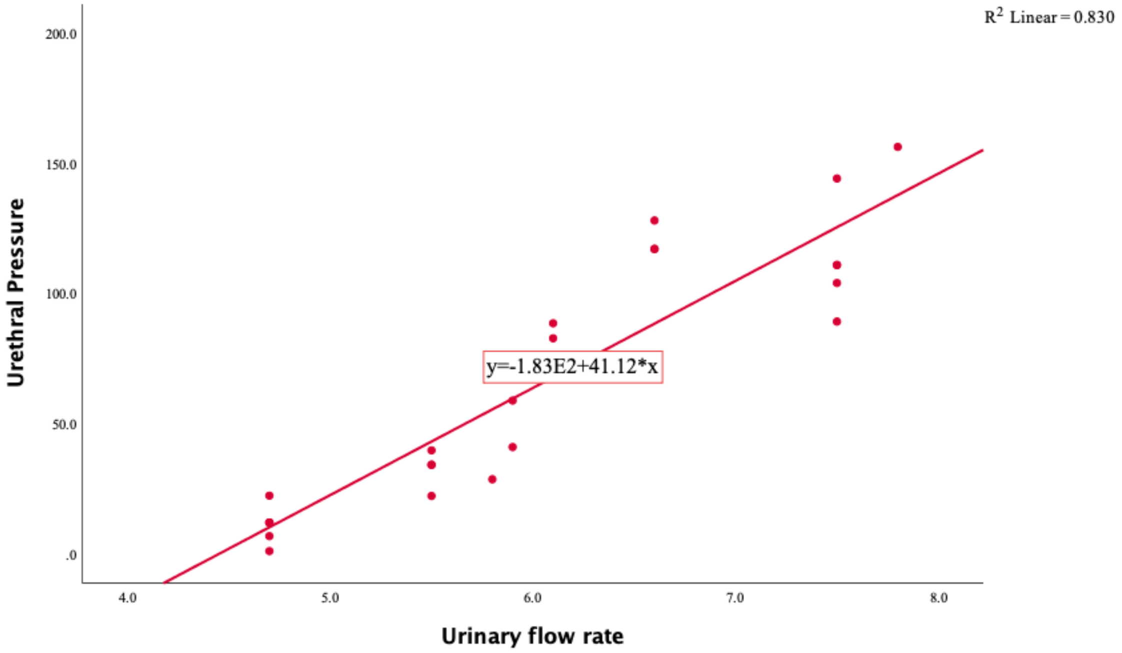

Fig. S3. The curve fitting results show a linear correlation between urethral pressure (cmH_2O) and urinary flow rate (ml/s).

Supplement materials S4. Derivation of urethral volume calculation in invasive measurement (no figure included)

According to the ideal gas equation: $P \times V = n \times R \times T$, where P is the pressure of the gas, V is the volume of the gas, n is the amount of substance of the gas, R is the ideal gas constant, and T is the temperature of the gas. Since the Jesus urethral compliance assessment device is a closed system with no gas entering or leaving during the measurement process, n is constant. The urethral pressure catheter is made of low thermal conductivity material, and the heat exchange effect due to air compression is negligible. Therefore, $P \times V = n \times R \times T = c'$ (constant), where $V = V_{urethra} + V_{syringe} + V_{tubing\ system}$.

Thus, $P_i \times (V_{urethra\ i} + V_{syringe\ i} + V_{tubing\ system}) = c'$

Calculated using the least squares method, we get $V_{tubing\ system} = 8.4$ml, thus, $c' \approx 197$

Therefore, $V_{urethra} = (197/P) - 8.4 - V_{syringe}$, and thus the urethral compliance can be calculated based on the change in urethral volume under pressure changes.
